# Genomic Characterization of an Emergent Cross‐Border Recombinant Duck Plague Virus With High Pathogenicity and Reduced Vaccine Efficacy in Guangxi, China

**DOI:** 10.1155/tbed/8379232

**Published:** 2026-08-10

**Authors:** Songlin Yang, Shijun Xu, Kairu Wang, Chao Liu, Hongchun Yang, Liuyang Yuan, Shuangshuang Ma, Huayan Xie, Zhimin Zhang, Gonghe Li, Hongbin Si, Kun Ning, Changbo Ou

**Affiliations:** ^1^ College of Animal Science and Technology, Guangxi University, Nanning 530004, China, gxu.edu.cn; ^2^ Qilu Animal Health Products Co. Ltd, Jinan 250100, China; ^3^ Medical Laboratory Animal Center, Tianjin University of Traditional Chinese Medicine, Tianjin 301617, China, tjutcm.edu.cn; ^4^ Guangxi Key Laboratory of Animal Reproduction Breeding and Disease Control, Nanning 530004, China; ^5^ Guangxi Zhuang Autonomous Region Engineering Research Center of Veterinary Biologics, Nanning 530004, China

**Keywords:** duck plague virus, pathogenicity, whole genome sequencing

## Abstract

Duck plague virus (DPV) is a major pathogen causing recurrent outbreaks in waterfowl. This study reports the comprehensive characterization of a novel DPV strain, designated GXGG240520, isolated from clinically diseased ducks in Guangxi, China. The strain was confirmed as a virulent DPV by PCR targeting the *UL2* gene (1002 bp) and exclusion of other common duck pathogens. In pathogenicity tests, ducks challenged with GXGG240520 exhibited severe clinical signs including anorexia, diarrhea, and hemorrhagic stools, with mortality concentrated between 5 and 9 days postchallenge and a final survival rate of only 10%. Pathological examination revealed hemorrhagic lesions in multiple organs and atrophy of immune organs such as the bursa of Fabricius and thymus. Viral load was highest in the thymus and lowest in the heart. Notably, challenge protection experiments demonstrated that the currently available vaccine (C‐KCE strain) provided only limited protection, with 60% mortality in vaccinated ducks, indicating reduced vaccine efficacy capability. Whole‐genome sequencing revealed a genome length of 162,188 bp with 78 predicted open reading frames (ORFs). Comparative genomic analysis identified 40 amino acid deletions and mutations across 26 ORFs. Homologous recombination analysis confirmed that GXGG240520 originated from cross‐border recombination between the Chinese virulent strain CHv (major parent) and the Bangladeshi strain YLBRDP 11 (minor parent). Furthermore, structure prediction of the UL47 protein revealed marked differences in *α*‐helix and *β*‐sheet organization compared to both parental strains. This recombinant strain with high pathogenicity and reduced vaccine efficacy potential poses a significant challenge to current DPV control strategies.

## 1. Introduction

Duck plague (DP), also known as duck virus enteritis (DVE), is an acute, febrile, and septic infectious disease affecting ducks, geese, and other Anseriformes birds [1, 2]. Initially reported in the Netherlands in 1923, DP subsequently emerged in countries such as the United States, Canada, and the United Kingdom and was first identified in China in 1957 [3–6]. Currently, DP remains highly prevalent in China, posing a serious threat to the local duck breeding industry and further exacerbating the economic burden on the sector [6, 7]. As a global disease, DP affects waterfowl across all ages and species, inflicting substantial economic losses on the global waterfowl farming industry due to its high mortality, strong pathogenicity, and reduction in egg production [8, 9]. Infected waterfowl typically exhibit clinical symptoms such as head swelling, anorexia, prostration, and photophobia, accompanied by varying degrees of hemorrhage in the liver and spleen, intestinal congestion, and hemorrhagic necrosis of the intestinal mucosa [10, 11].

DPV is an enveloped, spherical virus with a linear double‐stranded DNA genome, designated as classified under *Mardivirus anatidalpha1* within the genus *Mardivirus*, subfamily *Alphaherpesvirinae*, and family Orthoherpesviridae. Although DPV exists as a single serotype, strains isolated from different geographical regions may exhibit significant variations in both pathogenicity and genomic sequences [12]. Despite the existence of multiple DPV variants, all share a common genomic architecture consisting of a unique‐long (UL) region, a unique‐short (US) region, an internal repeat sequence (IRS), and a terminal repeat sequence (TRS), forming a UL–IRS–US–TRS genomic structure [13, 14]. DPV encodes 78 open reading frames (ORFs) for functional and structural proteins, with 65 ORFs located in the UL region, 11 ORFs in the US region, and the remaining 2 ORFs distributed between the IRS and TRS regions, respectively [15]. To date, currently, only a limited number of complete DPV genomes have been sequenced, including the Chinese virulent strain CHv (162,175 bp), the European virulent strain 2085 (160,649 bp), the Bangladeshi strain YLBRDP 11 (161,633 bp), and the Thailand strain DP7 (165,448 bp) [16–19].

DPV, belonging to the avian herpesviruses, is a herpesvirus that exclusively infects birds without affecting other animals. Avian herpesviruses possess five unique genes, namely, *LORF5* (*LORF11*), *LORF4* (*LORF9*), *LORF3*, *LORF2* (*vLIP*), and *SORF3*, which are implicated in viral virulence [20, 21]. Notably, the viral lipase encoded by *LORF2* can enhance the replication and pathogenicity of Marek’s disease virus (MDV) [22, 23]. However, the specific functions of these unique genes in DPV remain to be further elucidated.

In this study, we successfully isolated a strain of DPV from Guangxi, China, in 2024. Pathogenicity tests and whole‐genome sequencing analysis were conducted on the isolated strain, which provided a theoretical basis for the prevention of DPV in the Guangxi region and the development of corresponding vaccines.

## 2. Materials and Methods

### 2.1. Ethics Statement

All animal experiments were approved by the Animal Ethics Committee of Guangxi University (Approval No. GXU‐2024‐039) and performed in strict compliance with the institution’s guidelines on animal welfare. All ducks in this study were bred and cared for in accordance with humane procedures.

### 2.2. Sample Testing

Our laboratory received nine tissue samples from dead diseased ducks collected in Gangnan District, Guigang City, Guangxi. The samples were derived from two groups of ducks: the first group consisted of 41‐day‐old adult ducks that had emergent vaccination against DPV for 8 days, and the second group comprised 21‐day‐old ducklings with suspected DP, which had emergent vaccination with DPV vaccine for 6 days. After dissection, sterile scissors were used to collect the liver, spleen, and lung tissues from the carcasses. These tissues were then cut into small pieces, placed into 2 mL centrifuge tubes, and mixed with 1 mL of physiological saline and 2–3 sterile steel beads. The remaining tissue specimens were stored at −80°C for future use. The tissue samples were homogenized into tissue homogenates using a tissue homogenizer. The homogenates obtained were subjected to three freeze–thaw cycles at −80°C, followed by centrifugation at 4000×*g* for 10 min in a high‐speed centrifuge, and the supernatant was collected for subsequent experiments.

DNA was extracted using FastPure Viral DNA/RNA Mini Kit (Vazyme, China Nanjing). PCR was used to determine whether the virus was DPV. Primers were amplified by PCR using the primers listed in Supporting Information 1: Table S1. The target amplification fragment was 416 bp. The PCR cycling protocol included an initial denaturation at 95°C for 3 min, followed by 35 cycles of amplification consisting of denaturation at 95°C for 15 s, annealing at 60°C for 15 s, and extension at 72°C for 15 s, with a final extension at 72°C for 5 min. After PCR, 10 μL of the amplified products were analyzed by electrophoresis on a 1% agarose gel, followed by visualization using a gel imaging system and comparison of band sizes.

### 2.3. Isolation and Identification of DPV

Duck embryos were supplied by Guangxi Shilong Agriculture and Animal Husbandry Group Co., Ltd., China. The tissue homogenate supernatant was filtered through a 0.22 μm filter and then treated with a 1:100 dilution of 1000 IU/mL penicillin and streptomycin (Solarbio, Beijing, China) at 4°C for ~1 h to obtain the DPV viral suspension. This suspension was subsequently inoculated onto the allantoic membrane of 11‐day‐old duck embryos at a volume of 200 μL per embryo. Duck embryos that died within 24 h postinoculation were discarded, while those surviving to 72 h were collected and chilled at 4°C overnight to extract the allantoic fluid and allantoic membranes. These tallantoic membranes are placed into 2 mL centrifuge tubes and mixed with 1 mL of physiological saline and 2–3 sterile steel beads. The allantoic membranes were homogenized into tallantoic membranes homogenates using a tissue homogenizer. The allantoic fluid and tallantoic membranes homogenates underwent three freeze–thaw cycles at −80°C, followed by centrifugation at 5000×*g* for 10 min to collect the supernatant, which was then subjected to PCR analysis. Allantoic fluid samples testing positive for DPV were serially passaged twice in duck embryos. After three blind passages, DNA was extracted from the allantoic fluid and allantoic membranes was subjected to PCR analysis to confirm the successful isolation of DPV. According to the Chinese state standard, specific primers were used for the identification of DPV virulent strains and weak strains, with the target amplification fragment for virulent strains being 1019 bp and for weak strains being 491bp. DNA and RNA were individually extracted from the isolated duck plague virus (DPV). The SPARKscript Ⅱ RT Plus Kit (AG0304‐B, Sparkjade, China) was utilized for RT‐PCR and standard PCR to verify the viral specificity. Primers were amplified by PCR using the primers listed in Supporting Information 1: Table S1. After PCR and RT‐PCR amplification products were detected by 1% agarose gel electrophoresis, followed by visualization using a gel imaging system and comparison of band sizes [24, 25].

### 2.4. DPV Pathogenicity Test

Fifteen 1‐day‐old ducklings were supplied by Guangxi Shilong Agriculture and Animal Husbandry Group Co., Ltd., China. Nonvaccinated ducklings were randomly assigned to a challenge group (*n* = 10) and a negative control group (*n* = 5). Ducklings reared in wire cages with good ventilation and lighting. Throughout the entire experimental period, the ducklings were allowed to freely feed and water. At 21 days of age, ducks in the challenge group were inoculated via intramuscular injection in the leg with 100 μL of GXGG240520 viral suspension (3 × 10^5^ TCID_50_/100 μL). Conversely, the control group received an intramuscular injection of 100 μL of phosphate‐buffered saline (PBS) via the same route. The clinical symptoms of the experimental ducks were observed daily, and the mortality of each group was recorded. On day 11 postchallenge, all animals were euthanized by professional operators using cervical dislocation, and tissues such as the liver, spleen, and heart were aseptically collected. Tissue samples were processed for pathological examination and viral load detection using quantitative real‐time PCR (qRT‐PCR).

To assess the viral copy numbers in the collected tissues (heart, liver, spleen, lung, kidney, bursa of Fabricius, thymus, and cecum), the pathogenicity test were detected. qRT‐PCR was performed using ChamQ Universal SYBR qPCR Master Mix (Vazyme, Nanjing, China) to analyze the replication of the GXGG240520 isolate in each organ. Primers were amplified by standard PCR using the primers listed (Supporting Information 1: Table S1). The qRT‐PCR reaction conditions were as follows: pre‐denaturation at 95°C for 30 s; followed by 40 cycles of denaturation at 95°C for 10 s and annealing/extension at 60°C for 30 s; finally, melting curve analysis was conducted by slowly heating from 60 to 95°C. The absolute viral DNA copy number was calculated by qPCR using a standard curve generated, and all reactions were performed in triplicate.

### 2.5. Experimental Studies on the Protection of Animals Against the Isolated Virulent Strain Using Currently Available Vaccines

Thirty 1‐day‐old ducklings were supplied by Guangxi Shilong Agriculture and Animal Husbandry Group Co., Ltd., China. At 1 day of age, nonvaccinated ducklings were randomly divided into three groups: the challenge group GXGG240520 (*n* = 10), the vaccine group (*n* = 10), and the negative control group (*n* = 10). Meanwhile, the vaccine group of ducklings were intramuscularly inoculated with 250 μL of live DP vaccine (Jilin Zhengye Biological Technology Co., Ltd.) containing the chicken embryo‐attenuated strain of DPV (CVCC AV1222) via the leg muscle. A booster immunization with the same dose of the live DP vaccine was administered at 14 days of age to enhance immune responses. Serum antibody titers were monitored after the booster immunization to confirm whether the protective titer was achieved. Serum antibody levels were detected at 7 and 14 days postvaccination, and ducklings with antibody titers reaching the positive cutoff were considered immunologically successful. At 28 days of age, all immunologically successful ducklings from the vaccine group and all ducklings from the challenge group were intramuscularly inoculated with 100 μL of GXGG240520 viral suspension (3 × 10^5^ TCID_50_/100 μL) into the leg muscle. Concurrently, ducklings in the blank control group received 100 μL of PBS via the same route as a control. Throughout the entire experimental period, the ducklings were allowed to freely feed and water, the breeding conditions are consistent with those in the DPV pathogenicity experiment. Daily observations were conducted to record clinical signs, and mortality in each group was documented throughout the experimental period. On day 11 postchallenge, all animals were euthanized by professional operators using cervical dislocation.

### 2.6. Phylogenetic Analysis

To obtain and study the complete genome sequence of the DPV isolate GXGG240520, viral DNA was extracted and sent to Sangon Biotech (Shanghai) Co., Ltd. (Shanghai, China) for metagenomic sequencing via the second‐generation sequencing technology. SnapGene (Version 8.0) was used to predict ORFs and perform sequence alignment, which was regarded as the final assembly result of the complete genome of the isolate GXGG240520. The complete genome sequences of 22 reference DPV strains retrieved from the NCBI database were subjected to gene sequence alignment, homology analysis, and amino acid mutation site analysis using the MegAlign module of DNASTAR Lasergene 11 and MEGA (Version 11.0), and a phylogenetic tree was constructed. A neighbor‐joining (NJ) phylogenetic tree was constructed using the p‐distance model with 1000 bootstrap replicates.

### 2.7. Homologous Recombination

To explore potential recombination events of the GXGG240520 isolate, the complete genome sequence of this isolate was aligned with those of 12 reference DPV strains from the NCBI database using the Clustal W algorithm implemented in MEGA (Version 11.0). RDP (Version 5.1.0) was employed to screen for potential recombination events through seven methods, including RDP, GENECONV, BootScan, MaxChi, Chimaera, SiScan, and 3Seq. In RDP 5.1.0, a recombination DPV event was considered authentic if at least four detection methods detected recombination signals [26]. Further validation was conducted using Simplot (Version 3.5.1), where identity comparison was performed via the Simplot method with the window width and step size set to 200 bp and 20 bp, respectively. Based on these results, different genomic fragments were selected to construct phylogenetic trees to verify the outcomes of the recombination analysis.

### 2.8. Homology Modeling of the UL47 Protein

Based on the complete genome sequence of the GXGG240520 isolate, the amino acid sequences of the UL47 protein from the GXGG240520 isolate, the highly virulent Asian strain CHv, and the vaccine strain YLBRDP 11 were submitted to Google DeepMind’s AlphaFold3 for protein structure prediction [27]. The predicted structures of the UL47 proteins from these DPV strains were then visually compared and analyzed using PyMOL 2.6.0 [28]. Specifically, the differences in the number of *α*‐helices and *β*‐sheets among the predicted UL47 protein structures of the GXGG240520 isolate, the virulent CHv strain, and the YLBRDP 11 strain were examined and compared.

### 2.9. ARRIVE Statement

In this study, all experiments involving live animals were conducted in accordance with the ARRIVE guidelines.

### 2.10. Statistical Analysis

GraphPad Prism (Version 8.3.0) was used for statistical analysis, and the following symbols were used to indicate significant differences between groups:  ^∗^ *p*  < 0.05;  ^∗∗^ *p*  < 0.01;  ^∗∗∗^ *p*  < 0.001; and  ^∗∗∗∗^ *p*  < 0.0001.

## 3. Result

### 3.1. Identification of DPV Isolated From Clinical Samples

**Figure 1:**
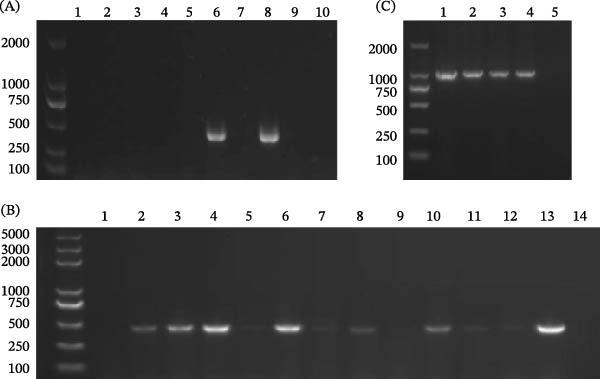
Isolation and identification of DPV isolates. (A) DPV was detected in Lane 6 and Lane 8. Target fragment: 416 bp PCR amplification; (B) DPV was isolated in Lanes 2–8 and 10−13. Target fragment: 416 bp PCR amplification; (C) The DPV was detected as a highly virulent strain. Target fragment: 1019 bp PCR amplification.

The results of pathological sample identification showed that the tissues of ducks No. 6 and No. 8 were confirmed to be DPV positive. PCR assays were conducted on the allantoic fluid and allantoic membranes of duck embryos inoculated with the DPV challenge virus solution (Figure 1A). The positive allantoic fluid was then passed through the duck embryos for two consecutive times, and the results indicated that DPV was successfully isolated, with electrophoretic bands consistent with the target size (Figure 1B). Primers for distinguishing virulent and attenuated strains were used to detect that the *UL2* gene of the DPV isolate was 1019 bp in length, identifying it as a DPV virulent strain (Figure 1C). The successfully isolated DPV was named GXGG240520 for subsequent experiments. The isolated DPV was tested for specificity with common duck pathogen diseases, including DPV, duck hepatitis A virus (DHAV), duck circovirus (DuCV), avian influenza virus (AIV), Muscovy duckling parvovirus (MDPV), duck Tembusu virus (DTMUV), fowl adenovirus (FAdV), Muscovy duck reovirus (MDRV), Newcastle disease virus (NDV), and duck astrovirus (DAstV). The test results showed that the DPV was positive and the other viruses were negative, confirming the successful isolation (Supporting Information 2: Figure S1).

### 3.2. High Pathogenicity of the DPV GXGG240520 Strain

**Figure 2:**
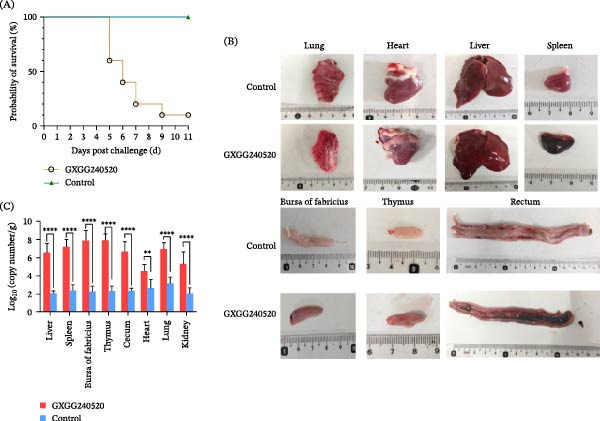
High pathogenicity of the GXGG240520 strain on ducks. (A) Survival rates of ducks injected with the GXGG240520 strain. (B) Clinical and pathological observations in ducks infected with the GXGG240520 strain (the control group is background noise). (C) Virus loads in different tissues of the infected ducks ( ^∗^ *p*  < 0.05;  ^∗∗^ *p*  < 0.01;  ^∗∗∗^ *p*  < 0.001;  ^∗∗∗∗^ *p* ≤ 0.0001).

To assess the pathogenicity and viral replication capacity of the GXGG240520 strain, animal experiments were conducted. Throughout the entire experimental period, the control group remained healthy and did not exhibit any clinical symptoms of DP. No significant differences were observed between the challenged group and the control group within 3 days postchallenge (dpc). However, on the 4th dpc, some ducks in the challenged group exhibited clinical symptoms, including lethargy, anorexia, ruffled feathers, and anal hemorrhage. Deaths in the challenged group began to occur on the 5th dpc, and the remaining ducks in the challenged group showed diarrhea, lacrimation, recumbency, mild head swelling, and some even presented with bloody stools. In addition, survival curves were plotted by recording the number of dead ducks using GraphPad Prism (Version 8.0.2). The analysis showed that the control group experienced no mortality throughout the experiment, maintaining a 100% survival rate. In contrast, deaths in the challenged group were mainly concentrated between 5 and 9 dpc, with a final survival rate of only 10% (Figure 2A).

Anatomical comparison between the control group and the experimental group revealed that in the challenged group, there were bleeding spots in the liver, lungs, and thymus. The spleen showed hemorrhagic enlargement, the bursa of Fabricius was atrophied, and the rectum had severe bleeding (Figure 2B).

Heart, liver, spleen, lung, kidney, bursa of Fabricius, thymus, and rectum samples were collected from each group of animals. The viral load of DPV in each tissue was detected, with the animals divided into a death group and a control group. Among the tissues, the viral loads in various tissues ranked from highest to lowest were as follows: thymus (8.7 × 10^7^ copies/g), bursa of Fabricius (7.3 × 10^7^ copies/g), spleen (1.4 × 10^7^ copies/g), lung (8.6 × 10^6^ copies/g), rectum (4.5 × 10^6^ copies/g), liver (3.7 × 10^6^ copies/g), kidney (2.0 × 10^5^ copies/g), and heart (3.3 × 10^4^copies/g) (Figure 2C). All experimental groups demonstrated significant differences in viral load compared to the control group, with the heart showing the smallest variation. These results indicate that DPV invades various tissues, causing varying degrees of damage.

### 3.3. The Currently Available Vaccine Provides Only Low Protection Against the Isolated Strain GXGG240520

**Figure 3:**
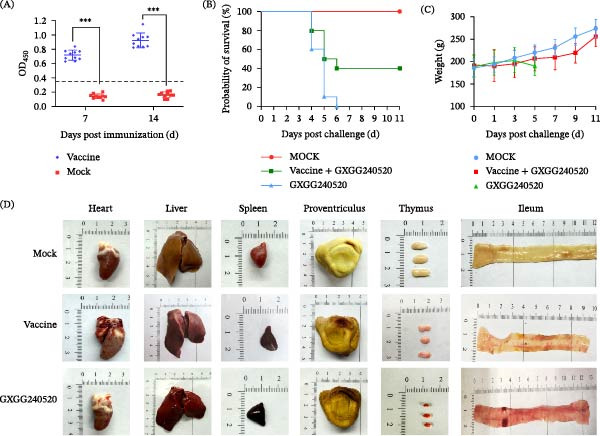
Current vaccine provides only low protection against the GXGG240520 strain. (A) Survival rate after GXGG240520 strain challenge. (B) Neutralizing antibody level detection after immunization AV1222 of ducks. Values above the dashed line are positive, those below are negative, and the dashed line represents the cut‐off value of 0.350. (C) Body weight change after GXGG240520 strain challenge ( ^∗∗∗^ *p*  < 0.001). (D) Clinical and pathological observations in immunized ducks and duck infected with the GXGG240520 strain.

To evaluate whether the current vaccine can effectively protect against the GXGG240520 strain, challenge protection experiments were performed in animal experiments. Blood samples were collected from all ducks in the challenged and vaccinated groups on days 7 and 14 after the booster immunization to isolate serum. The ducks were subsequently challenged with the DPV once antibody levels, as determined by ELISA, reached protective levels (Figure 3A). All ducks in the challenged group with GXGG240520 exhibited clinical symptoms such as recumbency and anorexia and succumbed to the infection within 4–7 days postchallenge. In the vaccinated group, deaths occurred between days 4 and 6 postchallenge, with a mortality rate of 60%, and the vaccinated ducks also showed symptoms including reduced appetite and depression, but no further deaths were observed after day 7 (Figure 3B). The body weight of ducks in the challenged group showed a declining trend postinoculation until all ducks died. All dead ducks were excluded from both groups, and only weight data of surviving ducks were analyzed. On this basis, the body weight variation trend of surviving ducks in the vaccinated group was similar to that of control group ducks (Figure 3C).

Tissue pathological examinations of the ducks revealed that the tissues from both the vaccine group and the challenge group exhibited differences when compared with those of the control group. Specifically, compared with the vaccine group, the liver and spleen in the challenge group showed pronounced hemorrhage and congestion. Additionally, the muscular stomach and thymus in the challenge group displayed atrophy along with hemorrhagic spots, and the intestine exhibited distinct circumferential hemorrhage (Figure 3D).

The results of the challenge protection experiments indicate that the current DPV vaccine strain C‐KCE (AV 1222) provides a certain degree of protection against the GXGG240520 strain; however, the GXGG240520 strain can still cause the death of most experimental animals.

### 3.4. Genomic Analysis of GXGG240520 With Multiple Amino Acid Site Mutations

**Figure 4:**
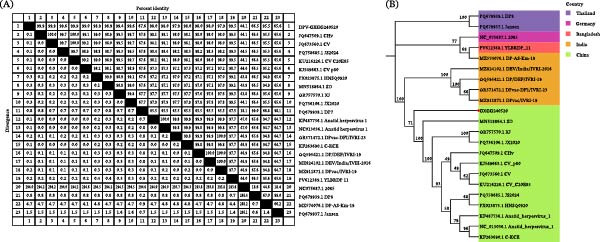
Genomic analysis of the GXGG240520 strain. (A) Homology analysis between the GXGG240520 strain and 22 reference strains was conducted using the parameter settings in Megalign, with the aim of examining the pairwise distances. The first row is the new strain GXGG240520. (B) Phylogenetic analysis of the GXGG240520 strain and reference 21 strains was performed. Phylogenetic trees were generated using the distance‐based, neighbor‐joining method implemented in the MEGA XI program. The reliability of the tree was assessed using the bootstrap analysis with 1000 replications. Red dot indicates GXGG240520 strain.

To elucidate the genetic basis underlying the high pathogenicity and diminished vaccine efficacy of the GXGG240520 strain, whole‐genome sequencing analysis were performed. The complete genome sequence of the GXGG240520 isolate comprises 162,188 nucleotides (bp), with a GC content of 44.8%. A total of 78 ORFs encoding proteins were predicted. Comparative and homology analyses were conducted between the GXGG240520 strain and 22 DPV whole‐genome sequences from Gene Bank, including DPV CHv and DPV CV. The results revealed that GXGG240520 is more closely related to the Asian virulent strains CHv and CV, with a high homology of 99.9% (Figure 4A). Phylogenetic tree analysis of the whole genome showed that the GXGG240520 isolate is positioned at the edge between Chinese strains and strains from other countries (Figure 4B).

Comparison of the UL2 gene of the GXGG240520 isolate indicated that its length is 1002 bp, consistent with the length of virulent DPV strains. To further analyze the differences among DPV strains, MegAlign was used to align the amino acid sequences of the ORF regions of 22 DPV strains. It was found that the GXGG240520 isolate has 26 ORFs in the UL, US, TRS, and IRS regions, with 40 amino acid site deletions and mutations that distinguish it from other strains (Table 1).

**Table 1:** Detection of amino acid mutation sites in 78 ORFs of GXGG240520. Forty amino acid mutations distributed across 26 ORFs.

ORF	Amino acid position(s)	Amino acid					
		GXGG240520	CHV (JQ647509.1)	CV (JQ673560.1)	YLBRDP 11 (PV612389.1)	C‐KCE (KF263690.1)	ELSE
UL56	78 148	**S→P**	**S→P**	S	S	S	S
		**G→S**	**G→S**	G	G	G	G
LORF5	348	**G→D**	**G→D**	G	G	G	G
	888	**R→C**	**R→** **C**	R	R	R	R
UL51	225	**P→Q**	P	P	P	P	P
UL49.5	79	**Y→F**	Y	Y	Y	Y	Y
UL47	148	^∗^ **D**	D	D	^∗^ **D**	D	D
	234	**P→S**	P	P	P	P	P
	727	**L→F**	L	L	L	L	L
UL41	126 166 172	^∗^ **D**	D	D	^∗^ **D**	D	D
		^∗^ **C**	^∗^ **C**	C	C	C	C
		**M→I**	M	M	M	M	M
UL38	385	**P→L**	P	P	P	P	P
UL37	5	**D→G**	D	D	**D→G**	D	D
	688	**T→N**	T	T	T	T	T
UL36	478 936 2207 2283	**T→A**	T	T	T	T	T
		**D→E**	D	D	**D→E**	D	D
		**T→A**	T	T	**T→A**	T	T
		**A→T**	A	A	A	A	A
UL30	452	**V→I**	V	V	V	V	V
UL29	1162	**M→V**	M	M	M	M	M
UL28	250	**L→V**	V	L	L	L	L
	256	**L→V**	V	L	L	L	L
	274	**R→S**	**R→S**	R	R	R	R
UL24	388	**T→S**	T	T	T	T	T
UL19	75	**L→F**	L	L	L	L	L
	921	**F→L**	F	F	F	F	F
UL12	368	**A→S**	A	A	A	A	A
UL8	733	**S→L**	S	S	S	S	S
UL6	281	**T→A**	T	T	T	T	T
UL3.5	14	**G→D**	G	G	G	G	G
UL3	113	**D→G**	**D→G**	**D→G**	D	D	D
IRS	1116	**N→T**	N	N	N	N	N
US1	5	**#Y**	—	—	—	—	—
US10	121	**P→S**	P	P	P	P	P
US3	182 331	**I→L**	I	I	I	I	I
		**Y→H**	Y	Y	Y	Y	Y
US7	5	**R→Q**	R	R	R	R	R
LORF3	25	**A→T**	T	A	A	A	A
TRS	1116	**N→T**	N	N	N	N	N

*Note:* Bold indicates the amino acid sites where mutations have occurred, and nonbold indicates the amino acid sites of the reference strain.  ^∗^ represents deletion. # represents addition, → mutation.

### 3.5. GXGG240520 Is a Recombinant Strain Derived From Highly Pathogenic DPVs of Chinese and Bangladeshi Origin

**Figure 5:**
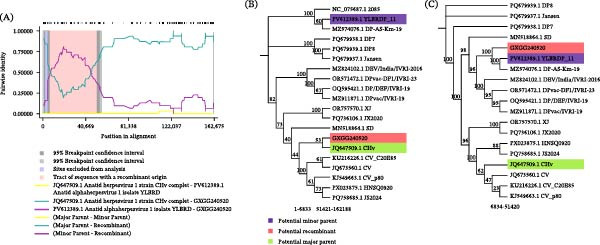
Recombination analysis of GXGG240520 isolate strain. (A) Detection of recombination events in GXGG240520 using RDP 5.10 software. The green line represents the pairwise identity between the major parental strain CHv and the isolate GXGG240520, and the red line represents that between the minor parental strain YLBRDP 11 and the isolate GXGG240520. The yellow line represents the pairwise sequence identity between the major parental strain CHv and the minor parental strain YLBRDP 11. (B) Regions with higher sequence identity to the major parental strain. Phylogenetic trees were constructed based on nucleotide sequences from different regions using the neighbor‐joining method in MEGA XI, with a Bootstrap value of 1000. The isolate is marked with a red rectangle, the major parental strain with a green rectangle, and the minor parental strain with a blue rectangle. (C) Regions with higher sequence identity to the minor parental strain. The isolate is marked with a red rectangle, the major parental strain with a green rectangle, and the minor parental strain with a blue rectangle.

The high number of amino acid mutations identified in the GXGG240520 genome prompted us to investigate whether this strain originated from genetic recombination between known DPV strains. GXGG240520 is a novel recombinant isolate of DPV generated from genetic recombination between strains CHv and YLBRDP 11 (Supporting Information 1: Table S2). Homologous recombination analysis was performed using the Recombination Detection Program (RDP) (Version 5.1.0), and among the seven analytical methods integrated in the software, five methods, namely, RDP, MaxChi, Chimaera, SiScan, and 3Seq, consistently confirmed that isolate GXGG240520 originated from a homologous recombination event, with strain CHv identified as the major parental strain and strain YLBRDP 11 as the minor parental strain of this recombinant isolate (Figure 5A). Nucleotide sequence homology analysis revealed that GXGG240520 shares 99.9% sequence identity with CHv in the regions spanning the regions of 1 to 6833 bp and from 51,421 to 162,188 bp. Within these regions, the phylogenetic tree analysis positioned the GXGG240520 isolated clustered closely with the virulent CHv strain, indicating a strong genetic relationship (Figure 5B). Conversely, in the region spanning from 6834 to 51420 bp, GXGG240520 exhibited a 99.9% sequence identity with YLBRDP 11. Correspondingly, in the phylogenetic tree for this region, the GXGG240520 isolate was closely clustered with YLBRDP 11, further substantiating the recombination event (Figure 5C). These findings collectively support the conclusion that GXGG240520 is a recombinant strain derived from CHv and YLBRDP 11.

### 3.6. The Protein Structural Differences of the UL47 Protein Between the GXGG240520 Strain and the Other Highly Virulent Strains

**Figure 6:**
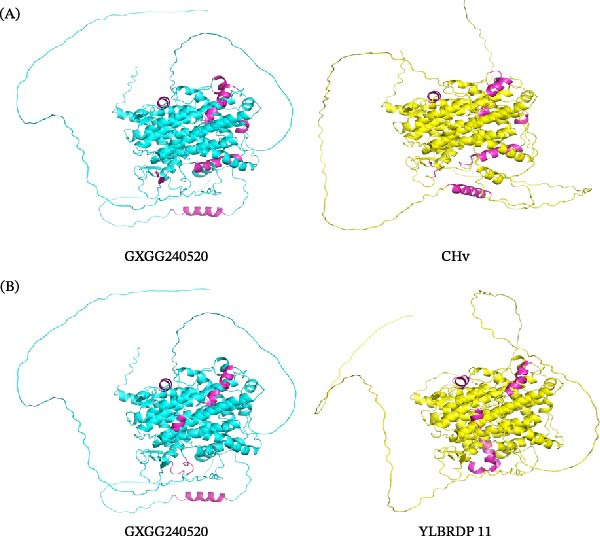
Comparative analysis of the UL47 protein between GXGG240520 and the other two highly virulent strains. (A) The purple region represents structural differences between the GXGG240520 strain of the blu and CHv strain of the yellow. (B) The purple region represents structural differences between the GXGG240520 strain of the blue and YLBRDP 11 strain of the yellow.

To determine whether the recombination event identified in GXGG240520 has resulted in structural alterations at the protein level, the UL47 protein structure was compared with those of its parental strains. By utilizing PyMOL 2.6.0 for visual comparison of the differences in the number of *α*‐helices and *β*‐sheets in the UL47 protein, significant disparities in the static structures of the UL47 among these strains were identified. Compared with the UL47 of CHv, the UL47 of the GXGG240520 isolate shows notable changes in its secondary structure. Specifically, one *α*‐helix was shortened by 4 residues. Additionally, a *β*‐sheet composed of three amino acid residues was added, while another *β*‐sheet of the same length was deleted. When compared with the minor parent YLBRDP 11, two *α*‐helices were shortened by 5 and 6 residues, respectively, two *α*‐helices of different lengths were added, and two *α*‐helices of different lengths were deleted (Figure 6).

## 4. Discussion

Based on the clinical presentation and pathological lesions consistent with DP, the causative agent was identified as DPV. This identification was subsequently confirmed through PCR detection, pathogen isolation and identification, whole‐genome sequencing, and animal pathogenicity tests. Specifically, the virus was isolated from 11‐day‐old duck embryos via allantoic cavity inoculation, leading to the successful isolation of the DPV strain GXGG240520 from Guangxi. The clinical manifestations of ducks infected with GXGG240520 closely resembled those previously reported, with an incubation period of ~2–3 days. Infected ducks exhibited anorexia, recumbency, reduced water intake, eyelid adhesion, and nasal discharge that thickened over time, eventually obstructing the nasal passages, with the highest mortality rate occurring 4–7 days postinfection. Pathological lesions were mainly observed in immune organs, including extensive hemorrhages in the liver, spleen, bursa of Fabricius, and thymus, along with varying degrees of atrophy and hemorrhage in the thymus and bursa of Fabricius, as well as severe hemorrhagic rings in the small intestine. DPV was detected in all tested organs, with the highest viral loads in the bursa of Fabricius and the thymus, followed by the liver. These findings suggest that the GXGG240520 isolate exhibits unique pathogenicity, preferentially replicating in immune organs, thereby compromising host immunity and leading to mortality.

The results of the challenge protection test revealed that the pathological change trends in the challenge group were highly consistent with those in the pathogenicity test. Experimental animals that were immunized and then challenged exhibited varying degrees of systemic hemorrhage, atrophy of immune organs, and a relatively high mortality rate. Although the extent of damage was less severe compared to that in the nonimmunized challenge group, this phenomenon still clearly indicated that the isolated strain had developed adaptability to the current vaccine and possessed reduced vaccine efficacy capabilities. This situation not only significantly increases the difficulty of using existing vaccines to control DPV but also brings new challenges and complexities to the in‐depth exploration of the pathogenic mechanism of DPV.

Whole‐genome sequence analysis of the GXGG240520 isolate revealed a genome length of 162,188 bp with a G + C content of 44.8%, indicating moderate codon stability. Bioinformatics predictions identified 78 ORFs in this isolation, with 40 amino acid mutations distributed across 26 ORFs. Notably, the UL19 gene, encoding the major capsid protein essential for viral replication, exhibited four amino acid mutations. Genes LORF11, UL47, UL41, UL24, and UL12 are associated with DPV virulence and involved in regulating viral replication, cellular dissemination, and virulence expression, all displayed amino acid mutations, warranting further investigation into their impact on viral transmissibility and pathogenicity [29, 30]. The US7 gene encodes glycoprotein I, whose deletion impairs viral envelope integrity and hinders virion maturation and intercellular transmission; the US3 gene encodes a serine/threonine kinase that regulates innate immune responses and apoptosis via substrate phosphorylation, playing a key role in DPV intercellular replication and virion release, and the impact of its nonsynonymous mutations on host cell infection and transmission efficiency merits in‐depth study [31–34].

pUL47 is a tegument protein encoded by the UL47 gene. As a virulence factor, it is abundantly present in virus particles. Although UL47 is a nonessential gene for virus replication, it is crucial for virus growth in vitro and plays an important role in intercellular viral spread and virus release, thereby facilitating viral production. Based on the functional attributes described above, pUL47 is consequently classified as a late structural protein. Deletion of the UL47 gene impairs the release of infectious mature viral particles and significantly reduces the virulence of DPV. DPV lacking UL47 has extremely high safety and can induce a high‐level antibody, thus providing complete protection for ducks, showing potential in vaccine development [23]. Moreover, the secondary structure of pUL47 in this isolate differs significantly from that of other isolates, with multiple nonsynonymous mutations at amino acid sites. These changes may lead to differences in intercellular spread efficiency and virus release between this isolate and other DPV isolates.

Homologous recombination analysis confirmed that the GXGG240520 isolate represents a recombinant strain derived from the Asian virulent strain CHv and the Bangladeshi virulent strain YLBRDP 11, first reported in 2023. Within the genomic regions exhibiting 99.9% nucleotide homology to the major parent CHv, 26 amino acid mutation sites were identified, with 9 mutations being consistent with those in CHv. Similarly, within the regions showing 99.9% nucleotide homology to YLBRDP 11, 13 amino acid mutation sites were detected, including 5 mutations that matched those in YLBRDP 11. These findings further substantiate the recombinant origin of the GXGG240520 isolate. This homologous recombination event suggests a risk of cross‐regional transmission for the isolate and potential immune evasion against existing live DPV vaccines in China, posing a potential threat to the current DPV control system [35]. Subsequent immunization protection trials demonstrated that while existing live DPV vaccines offer some protection against the isolate, the protection efficiency is significantly lower than that against other domestic DPV virulent strains, underscoring the necessity for in‐depth research into the isolate’s pathogenic mechanisms and tailored control strategies.

In summary, this study elucidated the ORF variations of the GXGG240520 isolate from Guangxi and its phylogenetic position relative to the reference strain. It was found that the isolate harbors numerous amino acid mutation sites, including a significant number of virulence genes. Additionally, the isolate exhibits certain adaptability and immune evasion toward existing vaccines, posing substantial challenges to the investigation of the pathogenic mechanisms of DPV and its prevention and control. Whether this immune evasion is associated with mutations in virulence genes requires further investigation. The potential for recombination among cross‐border strains also complicates efforts to control and understand the pathogenic mechanisms of DPV. Therefore, further research is crucial to determine how these amino acid mutations affect the pathogenicity and virulence of the isolate.

## Funding

The project is supported by the Guangxi Agriculture Research System Innovation Team (Grant nycytxgxcxtd‐2024‐19).

## Conflicts of Interest

The authors declare no conflicts of interest.

## Supporting Information

Additional supporting information can be found online in the Supporting Information section.

## Supporting information


**Supporting Information 1** Table S1:Primer sequences for RT‐PCR or qRT‐PCR in the paper. Supporting Information Table S2:The *P*‐values for the five recombination detection methods.

**Supporting Information 2** Figure S1:Monospecificity assessment of isolated virus. 1–2 duck plague virus (DPV), 3–4 duck hepatitis A virus type 1 (DHAV1), 5–6 duck hepatitis A virus type 3 (DHAV3), 7–8 duck circovirus (DuCV), 9–10 avian influenza (AIV), 11–12 Muscovy duckling parvovirus (MDPV), 13–14 duck Tembusu virus (DTMUV), 15–16 fowl adenovirus type 1 (FAdV‐1), 17–18 fowl adenovirus type 3 (FAdV‐3), 19–20 Muscovy duck reovirus (MDRV), 21–22 Newcastle disease virus (NDV), 23–24 duck astrovirus (DAstV).

## Data Availability

The data that support the findings of this study are openly available in Figshare at https://doi.org/10.6084/m9.figshare.31287646 [36]. All relevant data are within the paper and its Supporting Information files.
