# Supplementary material for: Genomic Characterization of an Emergent Cross‐Border Recombinant Duck Plague Virus With High Pathogenicity and Reduced Vaccine Efficacy in Guangxi, China
Source: Transbound Emerg Dis. 2026 Aug 10;2026:8379232. doi: 10.1155/tbed/8379232 (PMC13458276; doi:10.1155/tbed/8379232)
Supplement: Supplementary file 1 [file TBED-2026-8379232-s001.docx]

| Primer | Sequence(5’→3’) | exact national standard number | Country |
| --- | --- | --- | --- |
| DVEV P1 | GAG CGT ATT TAG TAG AAA CTG C | [GB/T 22332-2008](https://std.samr.gov.cn/gb/search/gbDetailed?id=71F772D75A54D3A7E05397BE0A0AB82A) | China |
| DVEV P2 | TGA ATG TTG TGA TTG TTC |  |  |
| DPV UL2-F | TAACGTCGTTTATACTGTTCCAC | DB35/T 2034-2021 | China |
| DPV UL2-R | AGACCCAAATGACAGAAACT |  |  |
| DPV-q-F | GTTTTGTGGGTGGGTCA |  |  |
| DPV-q-R | CAAGCCTATCCTCTTCGTC |  |  |
| DHAV-1 F | AAG AAG GAG AAA ATYCAAG GAA GG | NY/T 554-2023 | China |
| DHAV-1 R | TTG ATG TCA TAG CCC AAGACA GC |  |  |
| DHAV-3 F | TGG CTA TTG ACT TTG GCT T | NY/T 554-2023 | China |
| DHAV-3 R | TGT TAT GGA CTG GAA CCA CT |  |  |
| Ducv F | AAC CAC GCG GGA AGT GGT GGG | DB45/T 2103-2019 | China |
| Ducv R | TTC TAA GCA TAA ACG AGA TC |  |  |
| AIV M-U | TTCTAACCGAGGTCGAAAC | GB/T 18936-2025 | China |
| AIV M-L | AAGCGTCTACGCTGCAGTCC |  |  |
| AIV H5-R | GGAATATGGTAACTGCAACACCA | GB/T 18936-2025 | China |
| AIV H5-L | ACGGCCTCAAATTGAGTGTTCATTTT |  |  |
| AIV H7-R | AATGCACARGGAGGAGGAACT | GB/T 18936-2025 | China |
| AIV H7-L | TGAYGCCCCGAAGCTAAACCA |  |  |
| AIV H9-R | TGTGTCTTACGATGGGACAAGCA | GB/T 18936-2025 | China |
| AIV H9-L | TTGACAAGAGGCCTTGGTCCTAT |  |  |
| DPV-decF | AACTTACCAAGGCCCGTTCCTG | SN/T 5669-2024 | China |
| DPV-decR | GCGACGCTGTCCGCCTTGTTGA |  |  |
| DTMUV-F | AGACTGCTGGTGCAATGAAAC | NY/T 3233-2018 | China |
| DTMUV-R | CGTCGTTCCCAAGTTCCA |  |  |
| FAdV 1-F | GTGGTRTCCATGTTGGT | DB37/T 4754.3—2024 | China |
| FAdV 1-R | ATGTACGCYTCCGCCCTC |  |  |
| FAdV 3-F | CTGGTGAGTCTAGCTTGTCT | DB37/T 4754.3—2024 | China |
| FAdV 3-R | CACAATCAGTAGCAACACC |  |  |
| NDV F | ATGGGCCCCAGACCTTCTAC | GB/T 16550-2020 | China |
| NDV R | CTGCCACTGCTAGTTGTGATAATCC |  |  |
| DAstV F | ATTCTTGGCTCGGTTGTC | GB/T 45093-2024 | China |
| DAstV R | CCTGTGTTGCTCCTTCTC |  |  |

Supplementary Table
Table S1 Sequence of primers used to in the present study

| Recombinant Sequence | Major parent | Minor parent | Breakpoints (nt) | | Detection methods (*p* value) |
| --- | --- | --- | --- | --- | --- |
|  |  |  | Beginning | Ending |  |
| GXGG240520 | JQ647509.1 CHv | PV612389.1 YLBRD | 6834 | 51420 | RDP (9.29×10^-12^);  MaxChi (1.47×10^-3^);  Chimacra (4.49×10^-5^);  SiScan (1.16×10^-8^);  3Seq (1.72×10^-9^) |

Table S2 Recombination breakpoints identified in GXGG240520 strain and associated

parental strains.
